# Emotional Processing and Attention Control Impairments in Children with Anxiety: An Integrative Review of Event-Related Potentials Findings

**DOI:** 10.3389/fpsyg.2016.00562

**Published:** 2016-05-03

**Authors:** Erika Wauthia, Mandy Rossignol

**Affiliations:** ^1^Service of Cognitive Psychology and Neuropsychology, University of MonsMons, Belgium; ^2^Fonds pour la Recherche en Sciences Humaines/Fonds National pour la Recherche ScientifiqueBrussels, Belgium

**Keywords:** Anxiety, P1, N2, P3, ERN, LPP, attentional control, emotion

## Abstract

Anxiety disorders in adults have been associated with biased processing of emotional information which may be due to a deficit in attentional control. This deficit leads to an hypervigilance and a selective attention toward threatening information. Event-related potentials (ERPs) have been used to study this topic in anxious adults. Similar biases have been reported in children with anxiety but researches investigating the ERPs components underpinning these biases are more scarce. However, the understanding of the neural correlates of attentional biases in anxious children seem quite important since they could play a role in the etiology and the maintenance of this disorder. This review summarizes the results of researches having used ERPs to index emotional processing and attention control in children suffering from anxiety. We will focus on the P1, indexing basic visual perceptual processing, the N2, thought to reflect cognitive control process, the P3 typically associated with response inhibition, and the late positive potential (LPP) that indicates sustained attention toward motivationally salient stimuli. We will also examine the error-related negativity (ERN) that indexes monitoring system for detecting errors. Electro-physiological studies generally reported increased amplitudes of these components in anxious children, even when they did not differ from typically developing children at a behavioral level. These results suggest diminished cognitive control that influences children's selective attention mechanisms toward threatening information. Theoretical perspectives and implications for future researches will be discussed in the framework of current models of childhood anxiety.

## Introduction

Anxiety is a frequent and incapacitating psychiatric disorder that affects 15–20% of children and adolescents (for a discussion on the prevalence and the definition of anxiety in youths, see Beesdo et al., 2009). Pathological anxiety is defined by persistent or extensive anxiety and avoidance behavior, associated with subjective distress and impairments in daily life. These features make it possible to distinguish pathological anxiety from normative and developmental fears and concerns that often emerge during childhood and adolescence, but which are transient and do not interfere with the functioning of youths (Beesdo et al., 2009). Anxiety disorders in children ranges from high temperamental trait anxiety to clinical anxiety disorders, including separation anxiety disorder (SEP), social anxiety disorder (SAD), generalized anxiety disorder (GAD), panic disorder (PD), and agoraphobia disorder (AD) (Beesdo et al., 2009). Without intervention, anxiety is often a lifelong problem that persists into adulthood and significantly affects personal and professional achievements (Beesdo et al., 2009).

Anxiety significantly impairs cognition and anxious individuals consistently show a biased cognitive processing of emotional information (for a review, see Bar-Haim et al., 2007). Biases in the processing of emotional stimuli, such as threatening words or angry faces, have been reported in healthy children with a high level of temperamental trait anxiety and in children diagnosed with clinical anxiety disorders (for a review, see Puliafico and Kendall, 2006), suggesting that anxiety acts as an on/off filter in the cognitive processing of emotional information. As cognitive biases are postulated to play a role in the etiology and maintenance of anxiety, researchers have attempted to defined the exact stages of information processing that are affected by anxiety. Notably, Richards et al. (2014) distinguished between hypervigilance to threat, corresponding to the fast selection of threatening information in the environment, and selective attention to threat, which refers to the preferential processing of this information.

Given their high temporal resolution, event-related potentials (ERPs) constitute a very suitable method for exploring the dynamics of emotional processing (for a review, see Olofsson et al., 2008; Carretié, 2014). Numerous studies in anxious adults have demonstrated elevated amplitudes of early and late parietal components (Holmes et al., 2009; Michalowski et al., 2009; Mueller et al., 2009; Rossignol et al., 2012). However, studies in children are scarce and, to our knowledge, there has been no attempt to date to integrate the existing findings.

Accordingly, a first aim of this review is to summarize results stemming from electrophysiological studies that have explored ERP correlates of attention to threat in children suffering from clinical anxiety disorders and subclinical anxiety. A second aim of this review will be to discuss the role of attention and particularly attentional control in these biases. Indeed, contemporary models of attention postulate the coexistence of two neural systems, namely an automatic, stimulus-driven attentional system, and a more strategic, goal-directed attentional system (Posner and Petersen, 1990; Corbetta and Shulman, 2002). The balance between these two systems is ensured by attentional control (Eysenck et al., 2007). According to the Attention Control Theory (ACT, Eysenck et al., 2007), anxiety disrupts attentional control, leading to orientation of attention toward threatening but irrelevant events in a bottom-up manner, and disables the inhibition of such processing in a top-down manner. The hypothesis of an impoverished attention control in high anxiety has been supported by research showing reduced activity of the prefrontal cortex (PFC) (Bishop, 2009) and disrupted attentional control during emotional processing (Ansari and Derakshan, 2011a,b; Osinsky et al., 2012). Consistently, Telzer et al. (2008) have shown that adolescents (11–18 years-old) with a high level of anxiety demonstrate a selective attention bias toward angry faces associated with an enhanced recruitment of the right dorsolateral PFC, suggesting that that high anxiety requires increased cognitive control to reorient attention after processing threatening cues. Accordingly, an interesting question concerns the efficiency of attentional control in children and its role in the emergence of cognitive biases, since this process gradually develops during childhood. According to Lonigan and Phillips (2001), low effortful control abilities mediate the development of negative affectivity by shaping stimulus selection and then the subsequent cognitive and emotional processing (Lonigan and Vasey, 2009). Moreover, this model argues that children with high levels of negative affectivity demonstrate an automatic attentional bias for threat, but they may vary in their capacity to use voluntary attentional control to override this bias (Lonigan et al., 2004).

Hence, good attentional control may be protective against the development of pathological anxiety; it should therefore be important to examine the consistency of results between children suffering from subclinical and clinical anxiety, as well as in young children with high levels of shyness or behavioral inhibition (BI), described as a precursor of anxiety emergence (Van Ameringen et al., 1998; Muris et al., 2011).

Accordingly, this review will focus on five ERP components in order to evaluate their value in understanding the attentional biases present in children with anxiety. First, after the presentation of a visual stimulus, one may record the following component: (1) the P1, which indexes early automatic, exogenous attention (Carretié, 2014); (2) the N2, which is associated with conflict detection, and (3) the P3, reflecting motor control and response inhibition (Enriquez-Geppert et al., 2010; Luijten et al., 2014); (4) the LPP component, which indexes sustained attention toward motivationally salient information (Cuthbert et al., 2000). Finally, after the response production stage, (5) the error-related negativity (ERN) is also associated with response evaluation and cognitive control mechanisms (Hajcak et al., 2003). For each component, we will describe its development in childhood, its functions, and its relevance in studying emotional attention, before reviewing results stemming from empirical ERP studies (summarized in Table 1). Finally, we will discuss the implications for future research and theoretical perspectives in the framework of current models of childhood anxiety.

**Table 1:** **Results from empirical ERP studies in children with anxiety**.

**Reference**	**N**	**Diagnoses**	**Age**	**Questionnaires**	**Behavioral measures**	**Electrophysiological measures**	**Results**
Buss et al., 2011	35 (19♂)	Healthy controls	4- to 8-years-old	CBQ	The ANT test	EEG ERP (N2)	N2 effect in children older than 6 years old [*F*_(1, 11)_ = 9.05, *p* < 0.01] Increase in N2 was associated with less efficient executive attention and lower temperamental effortful control.
Carrasco et al., 2013	13 26 27	GAD and/or SAD and/or OCD OCD Healthy controls	8- to 16-years-old	K-SADS-PL CBCL CDI MASC	Eriksen Flanker Task	EEG ERP	Compared to healthy controls, ERN amplitude was increased in patients with either OCD or other anxiety disorders [*F*_(2, 62)_ = 7.16, *p* = 0.0016] Scored from the CBCL had a correlation with ERN amplitude in all subjects (*r* = −0.30, *p* = 0.013; *p* = −0.33, *p* = 0.007)
Davies et al., 2004	151 (89♀)	Healthy controls	7- to 18-years-old		Visual Flanker task	EEG EOG ERP (ERN, CRN, Pe)	ERN amplitude in error trials increased with age [*F*_(1, 122)_ = 20.9, *p* < 0.001] The Pe amplitude did not change with age. CRN amplitude was larger in children than in adults [*F*_(1, 116)_ = 8.6, *p* = 0.004]
DeCicco et al., 2012	34 (13♀)	Healthy controls	5- to 7-years-old	CBCL CBQ	IAPS The black box task	EEG EOG ERP (LPP)	Larger LPP amplitude for unpleasant pictures [*F*_(2, 60)_ = 11.85, *p* < 0.001] LPP was not sensitive to reappraisal [*t*_(31)_ = −0.80, *p* = 0.42]
Dennis and Hajcak, 2009	25 (13♀)	Healthy controls	5- to10-years-old	The Emotion Regulation Checklist CBCL	IAPS	EEG EOG ERP (LPP)	Smaller LPP following neutral interpretations at posterior recording sites, [*F*_(1, 16)_ = 7.93, *p* = 0.01) except for younger girls (aged 5–6 years) [*F*_(1, 16)_ = 5.32, *p* < 0.05]. Greater LPP modulation of the LPP by neutral interpretations was associated with reduced anxious-depressed symptoms (*r* = 0.49, *p* < 0.05)
Henderson, 2010	46 (24 ♀)	Extreme Shyness	9- to 13-years-old	EATQ-R SASC-R CASQ SPPS	Eriksen Flanker Task	EEG ERP (N2)	Shyness and N2 amplitude alone and in combination were associated with measures of social adjustment: -Negative attribution style: β = 0.66, *t* = −3.09, *p* = 0.004/β = -0.45, *t* = −2.24, *p* = 0.03) -Self-perception of social acceptance: β = 0.28, *t* = 2.24, *p* = 0.03 -Social anxiety: β = 0.89, *t* = 3.89, *p* < 0.001 Shyness was associated with larger N2 amplitude or enhanced N2 responses.
Hum et al., 2013a	29 34	Clinically anxious Typically developing	8- to 12-years-old	MASC CBCL STAIC-S	Go/NoGo	EEG ERP (P1, N2, ERN, CRN)	Greater P1 [*F*_(1, 57)_ = 5.56, *p* = 0.022) and N2 [*F*_(1, 57)_ = 9.48, *p* = 0.003] amplitudes in anxious children Greater ERN and CRN in anxious children [*F*_(1, 56)_ = 6.67, *p* = 0.012] No differences between faces types or trials in anxious children. N2 amplitudes for calm faces predict self-reported anxiety levels [*F*_(1, 61)_ = 5.16, *p* = 0.027]
Hum et al., 2013b	24 (8♂) 16 (7 ♂)	Anxious Non-anxious	8- to 12-years-old	MASC STAIC-S CBCL GIS	Go/NoGo	EEG ERP (P1, N2)	Greater P1 activation in non-improvers at both sessions (*p* < 0.022) Greater P1 amplitudes at pre-treatment predicts non-improvement following treatment (*p* = 0.030) Greater N2 activation for improvers at post-treatment (*p* = 0.043).
Jonkman et al., 2007	16 17 17	Healthy controls	6- to 7-years-old 9- to 10-years-old 19 to 23-years-old	CBCL	Go/no-go (CPT-AX task)	EEG EOG ERP (N2)	In children and adults, a bilateral source pair in the medial frontal cortex was involved in the generation of the N2. Children need and additional posterior source. In 6- to 7-year-old children, this posterior source was localized in the occipito-temporal areas. In 9- to 10-years-old children, the posterior sources shifted to parietal locations.
Kujawa et al., 2015	90 (41♂)	53 anxiety disorders (AD) 37 healthy controls (HC)	7- to 19-years-old	K-SADS PARS CDI	Emotional face-matching task	EEG EOG ERP (LPP)	Response accuracy: AD = HC (*p* > 0.46) Response time: AD = HC (*p* > 0.30) Enhanced LPP amplitude to threat in AD in late stage of processing (*p* = 0.03)
Ladouceur et al., 2006	19	9 anxious 10 control	11- to 12-years-old 12- to 16-years-old	K-SADS-PL CBCL SCARED CDI BDI	Visual Flanker task	EEG ERP (ERN, Pe)	Increased ERN amplitude in anxious children [*F*_(1.17)_ = 7.84, *p* < 0.05] Neural generators of the ERN in the anxious group is localized to the ACC. No group differences in Pe [*F*_(1, 17)_ = 0.25, *p* = 0.62]
Lahat et al., 2014	291 (125♂)	Behavioral inhibition (BI) Longitudinal study	4 months 24 and 36 months 7-years-old	CBCL SCARED-R K-SADS-PL TBAQ	Visual Flanker Task	EEG ERP (ERN/CRN, Pe)	Children with high BI displayed at age 7 years larger ERN amplitude than those with low BI [*F*_(1, 62)_ = 8.12, *p* < 0.01]
Lamm et al., 2014	108 (48♂)	Healthy controls	2- to 7-years-old	TBAQ CBQ CBCL	The Zoo game (Go/no-go task)	EEG ERP (N2)	BI was associated with increased performance accuracy, longer reaction times, greater N2 activation and higher estimated doral ACC and DLPFC activation.
Meyer et al., 2012	55 (31♂)	Anxiety disorders	8- to 13-years-old	Child SCARED Parent-SCARED	Visual Flanker Task	EEG ERP	Among older children (≥12.43- years old) a larger ERN was related to increased anxiety based on parent report (*r* = −0.35, β = −0.53, *t* = 2.69, *p* < 0.01).The relationship is opposite among younger children (*r* =0.23, β = 0.35, *t* = 1.67, *p* = 0.90)
Meyer et al., 2014	295 parents and children	Anxiety disorders	3-years-old	PSDQ	Go/No go task	EEG ERP (ERN and Pe)	Children with anxiety disorders are characterized by an increased ERN [*F*_(1, 294)_ = 6.13, *p* < 0.01 ERN amplitude is mediated by the relationship between harsh parenting and anxiety disorders in children.
Meyer et al., 2015	96	48 anxiety disorders 48 healthy controls	6-years-old	PAPA The Structured Clinical Interview for DSM-IV	Go/ No Go task	EEG ERP (ERN)	Larger ERN in anxious children [*F*_(1, 95)_ = 1.41, *p* = 0.24] Maternal history of anxiety disorder was associated with a smaller ERN [*F*_(1, 92)_ = 4.47, *p* < 0.05]
Moser et al., 2008	42	21 (15♀) high level-socially anxiety 21 (11♀) low level socially anxiety		SPIN DASS	Eriksen Flanker Task	EEG EOG ERP (N2, P3, LPP, CRN)	No group differences emerged on the behavioral measures Enhanced P3 amplitude for high socially anxious individuals [*F*_(1, 40)_ = 12.49, *p* = 0.001, *^2^ = 0.24] Enhanced P3 for threatening faces [*t*_(20)_ = 4.42, *p* < 0.001, *d* = 0.96]
Pollak and Tolley-Schell, 2003	28 (17♂)	14 physically abused 14 non-physically abused	8- to 11-year-old	PCCTS RCMAS CBCL	Selective attention paradigm	ERP (P1) EEG EOG	Enhanced P1 response to angry faces [*F*_(1.24)_ = 4,62; *p* < 0.05] Threatening cues affect the flexibility and the control of selective attention [*F*_(1, 2)_ = 4.42, *p* < 0.06]
Santesso et al., 2006	37 (16♂)	Healthy controls	10-years-old	CBCL	Visual Flanker Task	EEG ERP (ERN and Pe)	More reported OC behaviors were associated with larger ERN (*r* = −0.35, *p* = 0.02) and larger Pe amplitude (*r* = 0.43, *p* < 0.005) The more error that were committed the less pronounced the ERN (*r* = 0.46, *p* < 0.01) and Pe component (*r* = 0.33, *p* = 0.02)
Santesso et al., 2005	39 (16♂)	Control	10-years-old	JEPQR-S	Visual Flanker Task	EEG ERP (ERN and Pe)	High scores on the Psychoticism and low scores on the Lie scale were associated with smaller ERNs. Smaller ERNs are associated with committing more errors on incongruent trials (*r* = 0.41, *p* < 0.01)
Solomon et al., 2012	39 (22♂)	Healthy controls	5- to 7-years-old	CBQ CBCL	IAPS The black box task	EEG EOG ERP (LPP)	Larger LPP amplitude for pleasant and unpleasant stimuli Association between LPP amplitude for unpleasant stimuli and fearful behaviors (*r* = 0.38, *p* < 0.05)
Wiersema et al., 2007	13 children (7♂) 14 adolescents (9♂) 17 young adults (10♂)	Control	7- to 8-years-old 13- to 14-years-old 23- to 24-years-old	Abbreviated WISC-III and short version of the WAIS-III	Go/no-go task	EEG ERP (ERN and Pe)	Group did not differ in the ability to adjust to response strategies after making an error. The ERN amplitude increased with age [*F*_(2, 36)_ = 5.49, *p* < 0.008 The Pe amplitude did not change with age

## The P100

The P100, or P1, is an exogenous component elicited in extrastriate areas of the visual cortex when a visual stimulus is detected (Csibra et al., 2008). The P1 indexes basic visual perceptual processing and peaks approximately 90–110 ms after the occurrence of the stimulus (Allison et al., 1999; Sass et al., 2010). While the P1 indexes early, pre-attentive processes, it may be modulated by a top-down attention processes and its amplitude increases with stimuli appearing in an attended location (Luck et al., 2000). Moreover, P1 amplitude is enhanced when viewing emotional as compared to neutral faces, indicating that emotional load globally enhances basic visual processes (Batty and Taylor, 2003). While an attention preference for emotional, and particularly negative, faces seems to develop from the first year of life (Peltola et al., 2009; Hoehl and Striano, 2010; Jessen and Grossmann, 2016), few studies have examined the P1 in response to emotional stimuli in normatively developing preschool to school-aged children (Taylor et al., 2004; Batty and Taylor, 2006; Todd et al., 2008).

During an implicit processing task involving emotional faces, Batty and Taylor (2006) found that negative emotions (fear, disgust and sadness), in comparison to neutral or positive (happy and surprised) emotions, elicited later P1 latencies in 4- to 6-year-old children, but not in older children or adults, and postulated that young children rely on a rapid global processing for detecting emotion. This result was not replicated in a go/no-go study with children in the same age range, but those authors postulated that the faces used in their study may have been insufficiently disturbing to elicit emotional effect (Todd et al., 2008). Although inconsistent, these first results confirm the existence of a P1 response in preschool children, and emphasize the role of stimuli in modulating this brain response.

A second important result concerns the constant reduction of the P1 amplitude with age (Taylor et al., 2004; Batty and Taylor, 2006; MacNamara et al., 2016) which has been interpreted as indexing a reduction in cortical activity due to the increasing automaticity and efficiency of visual processing with age (MacNamara et al., 2016). Accordingly, the P1 amplitude may indicate the amount of cognitive resources devoted to processing a visual stimulus, with higher P1 indicating more attention and allocation of more cognitive resources.

Despite being of much interest, only few studies have investigated the P1 response in children with anxiety disorders. Preliminary results are described by Pollak and Tolley-Schell (2003), who recruited 8- to 11-year-old physically abused children. Maltreated children showed important levels of anxiety and biases in their abilities to recognize, express, and regulate their emotional states (Pollak et al., 2000). Pollak and Tolley-Schell (2003) used a selective attention paradigm where participants had to detect targets cued by emotional (angry and happy) faces. Behavioral results showed that maltreated children displayed hypervigilance toward angry faces, indicated by a faster response time (RT) for a target appearing in the location previously occupied by an angry face, and difficulties in disengaging from threat (i.e., a longer RT for targets appearing on the other side of the screen). Interestingly, physically abused children displayed enhanced P1 responses for angry faces, suggesting that threatening cues capture early attention and creates difficulty in control its allocation (Pollak and Tolley-Schell, 2003). More recently, Hum et al. (2013a) proposed a go/no-go task using angry, calm and happy expressions for typically developing children and clinically anxious children. They showed that, as compared to their age-matched peers, anxious children demonstrated greater P1 amplitudes in responses to faces, irrespective of their emotional content, showing heightened attention and/or arousal in response to these stimuli. The authors suggested that anxious children may have been more sensitive to the demands of the task, leading them to experience heightened arousal and to attribute more attentional resources to facial stimuli (Hum et al., 2013a).

A comparable hypothesis of general hypervigilance/ hyperarousal was proposed by Peschard et al. (2013) who reported enhanced P1 amplitudes in SAD adults while processing neutral and emotional faces, but also colored frames. Hence, anxiety could lead to an enhanced neural activation when performing experimental tasks. Unfortunately, the study by Hum et al. (2013a) did not allow attribution of the P1 enhancement to a particular type of anxiety disorders or even to a specific dimension of anxiety since participants in their study presented different types of anxiety disorders (GAD, SAD, and SEP) and comorbid anxiety disorders.

In a second study, Hum et al. (2013b) examined the changes induced by a cognitive behavioral therapy (CBT) program by measuring ERP responses before and after the intervention, in children presenting clinical anxiety disorders (SEP, SAD, GAD). They showed that higher P1 amplitudes at pre-treatment predicted non-improvement after the therapy. Moreover, children who did not improve in terms of anxiety levels after treatment had greater P1 amplitudes in both sessions, in comparison to children for whom anxiety levels decreased after treatment (Hum et al., 2013b). Unfortunately, this study encountered the same limitations as the previous one, as participants with various and comorbid anxiety disorders were recruited. Moreover, some children participating in the CBT protocol also had other pediatric conditions, such as ADHD, which may have reduced their response to a therapy targeting anxiety. Nevertheless, one may conclude that these results suggest that enhanced perceptual vigilance, representing heightened attention and arousal processes, limits the response to CBT strategies that are focused on more explicit strategies. Accordingly, it would be interesting to evaluate if P1 can be enhanced by a retraining of the attention processes, as proposed in the Attention-Bias Modification (ABM) programs that has been shown to reduce attention biases and anxiety symptoms in anxious children (Bar-Haim et al., 2011).

## The N200

The N200 or N2 is a fronto-central negativity observed at 200–300 ms post-stimulus which is generated by frontal structures, such as the anterior cingulate cortex (ACC) and the orbito-frontal cortex (Banich et al., 2001; Nieuwenhuis et al., 2003). The N2 is thought to index conflict monitoring and its amplitude reflects the extent to which attentional control is required to resolve conflict and inhibit incorrect responses (Van Veen and Carter, 2002; Nieuwenhuis et al., 2003; Dennis and Chen, 2007). The N2 is evoked during tasks in which two or more incompatible response tendencies are activated simultaneously, requiring the inhibition of a pre-potent response, or including incongruent stimuli, such as go/no-go or Flanker tasks. Thus, the N2 is linked to effortful control, i.e., individual differences in the ability to engage executive processes to inhibit dominant responses (Posner and Rothbart, 2007).

From a developmental perspective, the N2 is already seen at the age of 4 in the context of cognitive emotional challenges (Nelson and Nugent, 1990; Todd et al., 2008). The N2 amplitude is larger in young children, particularly under conditions that require cognitive control (Lamm et al., 2006; Todd et al., 2008; Buss et al., 2011) and decreases with age. This decline correlates with better achievement in cognitive tasks (Lewis and Stieben, 2004; Henderson, 2010). Henderson (2010) demonstrated that the age-related changes in the N2 components are due to the ongoing development of the ACC and the PFC. Moreover, the structures involved in cognitive control processes evolve with time. Using a go/no-go task, Jonkman et al. (2007) identified a bilateral source pair in the medial frontal cortex that was involved in no-go N2 activity in both children and adults. However, an additional posterior source was needed to explain the N2 distribution in children. This posterior source was localized in occipito-temporal areas in 6- and 7-year-old children, to shifting to parietal locations in 9- and 10-year-olds. The additional activation of posterior sources in the youngest children might indicate that executive control performance is less automatic or requires more effortful and attentional control (Jonkman et al., 2007). Recently, Buss et al. (2011) suggested that the N2 is a good biomarker for conflict-monitoring efficiency for children older than 6 years, who show larger N2 amplitude to incongruent compared to congruent flankers, but not in preschool-aged children. Indeed, after controlling for age, the N2 enhancement was associated with less efficient executive attention and a resource depletion that corresponded to interference with executive attention interference (Buss et al., 2011). To summarize, studies have considered enhanced N2 amplitude as reflecting less efficient monitoring of cognitive and emotional conflict.

In line with this concept, enhanced N2 amplitudes have been reported in conditions that are precursors to of anxiety disorders. In a first study, Henderson (2010) showed that shyness was unrelated to behavioral performances or ERP measures in a sample of typically developing 9- to 13-year-old children when performing an Eriksen Flanker task, but an extremely shy temperament in combination with larger N2 amplitudes predicted social anxiety outcomes. These results suggest that conflict sensitivity may alter the regulation of attention and emotion and reinforce the negative influence of temperamental factors. Recently, Lamm et al. (2014) observed that early reports of BI (at age 2 or 3) was associated with social reticence in 7-year-old children who demonstrated enhanced N2 amplitudes and higher activation of the ACC and the dorsal lateral PFC for no-go trials. Although these results did not allow a conclusion as to whether N2 activation results from social reticence or if it is a predictive factor for social reticence, they do suggest that a high level of control-related neural activation in childhood may be a biomarker for future anxiety symptoms, particularly in conjunction with an inhibited temperament.

Results in clinically anxious children are more controversial. Hum et al. (2013a) showed that anxious children had greater frontal N2 amplitudes to faces showing all emotions, while age-matched peers showed enhanced N2 amplitudes specifically for angry faces. Moreover, the differences observed in neural activation were not due to variations in task performance since anxious and non-anxious children had comparable behavioral performances (Hum et al., 2013a). Hum et al. (2013a) proposed that this enhancement indicated heightened neural activation due to emotional regulation processes, either because all emotional faces were appraised as threatening by anxious children, or because they felt anxious throughout the experiment and were less sensitive to the actual emotion load of the stimuli. Interestingly, more negative N2 amplitudes for neutral faces predicted increased self-reported anxiety, consistent with the idea that neutral faces may be perceived as threatening in anxious individuals (Hum et al., 2013a). However, in a second study using the same paradigm in children of the same age, the authors did not replicate their finding and found that anxious children did not have greater N2 amplitudes relative to the comparison group (Hum et al., 2013b). Since this second study compared relatively small groups of participants with various types of anxiety disorders in the clinical groups, a lack of statistical power may be the reason for the lack of replication, but the existence of the N2 enhancement in anxiety states should be confirmed in future studies. More disturbing, in their second study in which they measured ERP responses before and after a CBT treatment, Hum et al. (2013b) reported an increase in N2 amplitude from pre- to post-treatment in those who improved.

## The P300

The P300 or P3 is a positive component that peaks 300–500 ms after stimulus onset at parietal and midline scalp sites (Segalowitz et al., 2010; Hajcak et al., 2013). The PFC seems to be the primary generator of the P3 (Halgren et al., 1998). Classically, the P3 is measured in go/no-go conditions (Bokura et al., 2001). The literature has associated the P3 with response inhibition (Falkenstein et al., 1999; Righi et al., 2009; Enriquez-Geppert et al., 2010). In go conditions, the P3 is maximal at centro-parietal sites whereas in no-go conditions, the P3 is maximal at fronto-central sites (Fallgatter and Strik, 1999; Weisbrod et al., 2000). Weisbrod et al. (2000) showed that the P3 was lateralized to the left side of the PFC during no-go trials highlighting the importance of this area in attentional control. In this review, we will mainly focus on the no-go-P3.

Bruin et al. (2001) showed that the P3 amplitude was enhanced when responses required stronger inhibition. The P3 follows a slow developmental course in childhood. Davies et al. (2004) used ERPs to investigate the response inhibition through the P3 in 6-year-old children, in comparison to a group of adults. During this task, participants performed a task that required selective responses to target stimuli while inhibiting responses to equally salient non-target stimuli. The results of Davies et al. (2004) showed that the neural activity measured by P3 differed between adults and children, suggesting that they may use different processes to perform an inhibition task (Davies et al., 2004).

Using a go/no-go task, Jonkman et al. (2003) compared the performance and ERP activity of 9- to 10-year-old children with those of adults. They observed that, in comparison to adults, children showed reduced or absent frontal P3, which would be due to an immature response inhibition processing in middle childhood (Jonkman et al., 2003). From a behavioral point of view, significant age effects were observed on hits, false alarms, impulsivity and inattention scores. Indeed, adults had more hits and less false alarms than children and were less impulsive and inattentive (Jonkman et al., 2003). Jonkman (2006) wanted to extend these results on neurocognitive development of the response inhibition measured by the P3 in a go/no-go task. Jonkman (2006) compared a group of 6- to 7-year-old to a group of 9- to 10-year-old and a group of adults of 19–23 year of age. The author observed a linear increase in the P3 effect with age. Indeed, a small effect was observed in late childhood at the midline frontal-central electrodes, but not in 6- to 7-years-olds. Furthermore, after 11-years-old, the P3 was recorded at frontal electrodes (Jonkman, 2006). These studies highlighted the fact that the P3 has a late development starting at about 10 years of age and are in agreement with previous studies (Jonkman et al., 2003; Okazaki et al., 2004). These data can be related to the late development of the networks involved in the regulation of motor preparation and response inhibition (Jonkman, 2006) and with the increase of the cortical efficiency with age (Lewis et al., 2006).

While the P3 presents a major interest in studies of motor response preparation (in go trials) and inhibition (in no-go trials), only few studies have measured this component in children with anxiety. Some preliminary results may be found in the study by Shackman et al. (2007), who, using a modified recognition oddball task, investigated how physically abused children process conflicting visual and emotion cues posed by their mothers or a stranger. They found that abused children showed enhanced P3 amplitudes in response to anger expressed by their own mother. Authors concluded that abused children exert more cognitive effort both to engage their attention toward salient anger cues and concurrently to inhibit the processing of irrelevant affective cues in the environment.

Éismont et al. (2009) examined the characteristics of amplitudes and frequencies of ERPs in 10- to 11-year-old children suffering from low and high anxiety levels with a two-stimulus go/no-go paradigm. They showed that the P3 wave for children with high anxiety levels were lower than for those of the same age with low-anxiety levels, suggesting that there are particularities in the functioning of the cerebral systems in anxious individuals. These authors supported the idea that decreased EP amplitudes in high anxiety children reflect insufficient maturity and unbalanced functioning of the brain structures (Éismont et al., 2009).

## The late positive potential (LPP)

The late positive potential (LPP) is a slow positive wave appearing at midline parietal sites at 400–600 ms after the onset of a relevant stimulus (Cuthbert et al., 2000). The LPP reflects sustained attention toward motivationally salient information (Kujawa et al., 2015). It has been proposed that the LPP reflects the same mental processes as the P3 (Kok, 1997) but unlike the P3, the LPP has been shown to be sustained throughout and even after picture presentation (Cuthbert et al., 2000; Hajcak and Olvet, 2008). Furthermore, the scalp topography is different between the LPP and the P3. Indeed, there is a scalp shift in the LPP from a parietal positivity in the 100–1000 ms range to a more superior positivity in the 1000–2000 ms range suggesting that the LPP is a slow wave (Hajcak et al., 2007; Foti and Hajcak, 2008) whereas the P3 is a peak (Foti et al., 2009). Furthermore, Foti et al. (2009) showed that the LPP reflects emotionally relevant processing, which is distinct from the P3. The LPP has been demonstrated to be associated with the activation of the visual cortex, as well as subcortical structures including the amygdala, ventral striatum, ACC and anterior insula (Liu et al., 2012).

The LPP has been demonstrated to be larger for emotional, both pleasant and unpleasant, stimuli (Cuthbert et al., 2000). Using an emotional face-matching task, MacNamara et al. (2013) has demonstrated that the LPP is larger for pictures of faces than of geometric shapes. Previously, Grasso and Simons (2012) has demonstrated that personally salient social stimuli, such as pictures of a relative, also elicit larger LPPs. Finally, the LPP has been demonstrated to be larger for stimuli described as negative in comparison to stimuli described as neutral (MacNamara et al., 2009).

In children, the LPP appears maximal at slightly more occipital recording sites as compared to adults, and was not evident in the ERP beyond 1500 ms. Recently, Kujawa et al. (2012) studied the electro-cortical reactivity to emotional faces in a sample of 3- to 6-year-old children and showed that the LPP arose in response to emotional faces in children who were only 6 years of age. Three studies evaluated the LPP in response to emotional pictures from the International Affective Picture System (IAPS, Lang and Bradley, 2007). First, Solomon et al. (2012) examined whether the LPP is sensitive to emotional content in healthy young children and whether this varies with affective individual differences. To do so, they measured neural responses to 30 unpleasant pictures (e.g., air crashes, snakes), 30 neutral pictures (e.g., household objects) and 30 pleasant pictures (e.g., Disneyworld, ice-cream) in 5- to 7-years-old children. They found that children showed larger LPP amplitudes for emotional stimuli. Second, Hajcak and Dennis (2009) confronted 5- to 8-year-olds with developmentally appropriate pictures and they recorded an increased amplitude of the LPP in response to unpleasant pictures. The differences in these studies may be partly due to the arousal by these stimuli. Indeed, Hajcak and Dennis (2009) found that pleasant stimuli were rated as more arousing than unpleasant stimuli in 5- to 10-years-olds. However, in the study by Solomon et al. (2012), the results can be interpreted as indicating the increased effort needed to interpret neutral facial expressions, because they are perceived as ambiguous and that this ambiguity is thought to convey emotion in anxious children.

Finally, Kujawa et al. (2012) found larger LPPs to emotional and neutral scenes in 8-to 10-year-olds than in a group of 11-to 10-year-old children. According to the authors, the age-related decrease suggests a shift in attentional allocation and stimulus processing which may be due to maturation of the cortical structures (Kujawa et al., 2012). In another study, these authors investigated the same children 2 years later, and confirmed the decrease of the LPP amplitude over time (Kujawa et al., 2013). MacNamara et al. (2016) replicated these finding while studying age-related change in ERPs elicited by emotional faces across an age span of 7- to 19-years-old. Kujawa et al. (2013) also investigated the stability of the LPP across development. They assessed this component during an emotional-interruption task following pleasant, unpleasant and neutral images in 8-to 13-year-old children that the results indicated that the LPP is quite reliable across ages. Thus, LPP appears to be a stable measure of emotional processing across a large period of development (Kujawa et al., 2013).

An important study by Kujawa et al. (2015) recruited children of 7-to 19-year-old with anxiety disorders who were exposed to an emotional face-matching task in which participants were shown fearful, happy or angry faces. Kujawa et al. (2015) observed a persistent enhancement of LPP amplitudes in the relatively late stages of processing (until 1000–2000 ms after the presentation of angry and fearful faces). Kujawa et al. (2015) compared the impact of different types of anxiety disorders on the LPP. They highlighted that LPPs to threatening faces were enhanced for SAD, compared to GAD or SEP. The results of this study suggest that the LPP may be a useful measure of threat reactivity in children with anxiety disorders and they confirmed the hypothesis of the early development of attentional biases toward threat in youths (Bar-Haim et al., 2007). The enhancement of the LPP in reaction to threats and particularly with subject-specific stimuli has also been demonstrated in adults suffering from anxiety disorders (Kujawa et al., 2015).

Moreover, higher LPP amplitudes to unpleasant stimuli were associated with longer RTs in a specific task called “the black box task” (Goldsmith and Rothbart, 1999). In this task, 5-to 7-year-old children were asked to put their hands inside a box that had “something scary inside” and the independent measure was the time required for the children to do so. These results suggest a relationship between a more positive LPP and fearful behaviors in children of these ages. Finally, given the high comorbidities between anxiety disorders and phobias (James et al., 2013), Leutgeb et al. (2010) studied the LPP in response to symptom provocation in 8- to 12-year-old spider-phobic girls, using a behavior avoidance test (BAT). Their results showed that phobic girls showed enhanced LPP amplitudes in response to spider pictures, reflecting motivated attention to emotionally salient stimuli.

The LPP can also be used to study the development and the maintenance of emotional regulation in anxiety disorders (Hajcak and Dennis, 2009). Interestingly, LPP amplitudes can be increased or attenuated if participants are asked to direct their attention to less or more arousing portions of emotional stimuli (Dunning and Hajcak, 2009) using a technique called reappraisal (Wessing et al., 2015). DeCicco et al. (2012) questioned reappraisal and LPP down-regulation in a group of 5- to 7-year-old children: the LPP amplitudes were larger in response to unpleasant than to neutral pictures and correlated with greater maternal-reported anxiety and observed fearful behaviors, but reappraisal did not modify LPP. The authors concluded that young children do not yet have the neural maturity to use reappraisal strategies effectively in order to regulate the affective and attentional processes measured by the LPP (DeCicco et al., 2012).

Consistently, Dennis and Hajcak (2009) recruited older children from 5 to 10 years and found that reappraisal led to a reduction of the LPP amplitude in response to IAPS stimuli (Dennis and Hajcak, 2009), supporting the idea that reappraisal capabilities evolve with the maturity of the fronto-parietal network (Wessing et al., 2015). Finally, Hua et al. (2015) examined changes in LPP amplitudes following simplified interpretations of unpleasant pictures in preschoolers. They showed that LPP amplitudes, after neutral interpretations, were lower than after negative interpretations, suggesting that preschoolers as young as 4 years have developed the ability to use cognitive reappraisal strategies following instructions.

Even though there have been no studies investigating the reappraisal abilities in anxious children to date, the empirical data presented here suggest that the LPP may be an useful measure of cognitive emotion regulation, threat reactivity and emotion processing biases in youths with anxiety, in relation to age.

## Component related to answer processing: the ERN and the Pe

The ERN is a negative deflection that occurs at fronto-central sites approximately 60–110 ms after a wrong response (Falkenstein et al., 1991, 2000; Luu et al., 2003; Pailing and Segalowitz, 2004). The concept of “errors” may refer to incorrect responses in choice-reaction time tasks (errors of choice) and uninhibited responses, for instance on no-go trials (errors of commission; Scheffers et al., 1996) or to the lack of an answer in a target trial (errors of inaction). The ERN is not affected by the type of the stimulus (Bernstein et al., 1995) or the modality in which the stimulus is presented (Falkenstein et al., 2000) and is output-independent (Holroyd et al., 1998). The ERP wave is considered to be a signal resulting from the mismatch between a response and the outcome of this response (Falkenstein et al., 2000; Wiersema et al., 2005). Taken together, the ERN reflects a “monitoring system” for detecting errors (Wiersema et al., 2005; Santesso et al., 2006). This monitoring system is essential for preventing undesirable actions and optimizing task performance (Wiersema et al., 2007).

The production of an error may also be followed by a second component, the error positivity (Pe, Falkenstein et al., 1991). This late positive deflection occurs at centro-parietal sites approximately 200–400 ms after response execution (Falkenstein et al., 2000; Ladouceur et al., 2006) and has been observed for corrected and uncorrected responses, as well as on false-alarm trials (Nieuwenhuis et al., 2001). Thus, the Pe cannot be considered as a correlate of the error correction process but is more likely to be a conscious process of the error event (Falkenstein et al., 2000). According to Falkenstein et al. (2000), the Pe may be related to the controlled adjustment of response strategies after the recognition of an error, or may reflect the recognition of the error (Wiersema et al., 2007). Furthermore, the Pe may reflect the emotional significance of the error to the participant (Santesso et al., 2006).

Error monitoring underlined by the ERN and the Pe is associated with activation in the ACC (Kiehl et al., 2000), which is centrally involved in controlling or directing attention and actions (Wiersema et al., 2007). However, the ERN and the Pe may be functionally and anatomically distinct (Wiersema et al., 2007) as studies have localized the generator of the Pe in the rostral part of the ACC and the parietal cortex whereas the ERN is generated in the caudal part of the ACC (Van Veen and Carter, 2002).

From a developmental point of view, the ability to identify error production seems to have matured at the age of 4 years and undergoes its maximal development during early adolescence (Davies et al., 2004; Ladouceur et al., 2004; Hogan et al., 2005; Grammer et al., 2014). Using a go/no-go task, Wiersema et al. (2007) found that latencies of incorrect responses compared to correct responses were shorter in children and adolescents than in adults. Indeed, they showed that children (age 7–8 years) have much smaller ERN than adolescents (age 13–14 years) and adults (age 23–24 years) and thus ERN seems to increase with age. The same effect was observed for ERN latencies: latencies for incorrect compared to correct responses were shorter in children and adolescents than in adults (Wiersema et al., 2007). These findings are in line with those of Davies et al. (2004), and those of Ladouceur et al. (2004), who did not find a difference between children (age 9–14 years) and adolescents (age 14–17 years) in ERN and Pe amplitudes and latencies. Taken together, these results suggest that the ERN and the Pe do not seem to appear until late adolescence suggesting that the ability to detect error-related conflict which involves the modulation of cognitive control, develops in adolescence (Ladouceur et al., 2004).

Anxiety disorders in children, similar to high levels of negative affect or depression, have been associated with increased ERN amplitudes resulting from altered maturational patterns of ACC circuitry (Ladouceur et al., 2006). Three studies used a visual flanker task to reveal higher ERN amplitudes in children with anxiety. Ladouceur et al. (2006) demonstrated increased ERN amplitude in 10- to 12-year-old anxious children as compared to children with no affective disorders, and attributed this result to higher levels of negative affect in anxious children. This result was replicated in 8- to 16-year-old children with obsessive–compulsive disorders, GAD and SEP (Carrasco et al., 2013). Moreover, Santesso et al. (2006) showed that parent-reported obsessive–compulsive behaviors were associated with larger ERN and Pe components at fronto-central sites in clinically normal 10 year-old children. Using a go/no-go task with facial stimuli depicting angry, neutral, and happy expressions, Hum et al. (2013a) confirmed that anxious children produced greater ERN amplitudes and thus greater error-related negativities and correct-response negativities than typically developing children. At a behavioral level, there was no significant differences in accuracy, response duration, go response times or error no-go response times, suggesting that the neural activation is not due to variation in task performance (Hum et al., 2013a).

The hypothesis of a relationship between negative affect and an enhanced ERN is congruent with the model developed by Luu et al. (2004). This model assumes that the ERN indexes an affective signal of distress when an individual detects a discrepancy between action and an emotionally-salient goal (Weinberg et al., 2012).

Interestingly, enhanced ERN amplitudes at age 6 years predicted the onset of anxiety disorder by age 9 (Meyer et al., 2015). Moreover, Meyer et al. (2012) found the relation between anxiety disorders and ERN amplitudes is also moderated by age: increased anxiety based on parent report was correlated to a larger ERN in older children (age 11–13 years), but this relation was opposite for younger children (age 8–10). These results indicate that ERN evolves with development to increase in anxious children around age 12 years (Davies et al., 2004).

Finally, the ERN appears to be modulated by early childhood temperament (Lahat et al., 2014). Using a visual Flanker task, these authors found that a high BI at age 7 years is associated with increased ERN amplitudes and predicts social phobia symptoms at age 9 years (Lahat et al., 2014). Increased ERN amplitude may index enhanced vigilance and fear of negative evaluation, and rigid and over-controlled behaviors, in social situations which, in turn, could lead to maladaptative social–emotional behaviors including phobia symptoms.

That relationship between a history of inhibited temperament and subsequent altered reward processing is congruent with the functional magnetic resonance (fMRI) results from Bar-Haim et al. (2009), who demonstrated heightened response monitoring and increased performance concerns in 14- to 18-year-old adolescents (Bar-Haim et al., 2009). The association between negative affect and the perceived distress with error commission may be reinforced by punitive parenting, as suggested in a longitudinal study by Meyer et al. which followed children over a period of 3 years (2013, 2014, 2015). These authors evaluated parenting behavior and parenting style in parents of 3-year-old children and they used a go/no-go task in a follow-up assessment, which included evaluation of child psychopathology 3 years later (Meyer et al., 2013, 2015). Moreover, in children, a larger ERN could be predicted by observational and self-reported harsh parenting, possibly because a hostile parenting style intensifies the threatening value and salience of errors and caused children to pay increased attention to their own error production, which is a risk for anxiety (Meyer et al., 2014).

In conclusion, although little information is available on Pe in children with anxiety, the ERN appears to be consistently modulated by anxiety, and could therefore constitute a promising biomarker for exploring defensive reactivity in children with anxiety symptoms or those who are at risk of anxiety.

## Discussion

The aims of this paper was to present the electro-physiological components underpinning the processing of emotional information and to discuss the role of an attentional control deficit in the development and maintenance of attentional biases in anxious children. First, we will present the results for each component of interest highlighted in this review.

The results of this paper showed that very few studies have investigated the automatic visual processing in anxious children and thus the P1. However, the studies that have investigated this component showed a larger P1 amplitude when anxious children were confronted with emotional face regardless of the emotional valence of the face. These results suggest an increased attentionnal processing of emotional faces in anxious children (Hum et al., 2013a). Thus, we can conclude that faces are considered as threatening by anxious children regardless of their emotional valence. Furthermore, there is a selective attention bias toward these faces (Richards et al., 2014) and the automatic attentional subsystem in anxious children would have superiority due to a deficit in attentional control (Eysenck et al., 2007). These results are in line with those observed in adults with high trait anxiety, who showed an enhancement of the P1 in response to angry faces (Fox et al., 2008; Frenkel and Bar-Haim, 2011), but also to phobia-relevant stimuli in phobic adults (Michalowski et al., 2009), and to all emotional stimuli in socially anxious adults (Mueller et al., 2009; Muhlberger et al., 2009; Rossignol et al., 2012) suggesting an increased vigilance in anxious states (Peschard et al., 2013). Given the results presented here, we can conclude that the P1 can be considered as an endophenotype of anxious disorders but also a biological marker useful for assessing treatment's efficacy.

We observed that the amplitude of the N2 can also be modulated by anxiety disorders (Hum et al., 2013a,b). Indeed, using a go/no-go task, Hum et al. (2013a) demonstrated larger N2 amplitudes in children who were confronted with emotional faces regardless of their emotional valence. These results suggest that anxious children allocate more neural resources to attentional control and inhibition. Furthermore, the lack of emotional specificity can be interpreted in two different ways: either anxious children consider every emotional face as threatening, or they feel anxious throughout the task and thus cannot distinguish different emotional patterns (Hum et al., 2013a). In their study, Hum et al. (2013a) highlighted that the neurophysiological data can be observed in the absence of behavioral effects. They confirmed the hypothesis of the attentional control theory developed by Eysenck et al. (2007). In their theory, Eysenck et al. (2007) differenciated the notions of efficiency and effectiveness, postulating that anxious individuals show similar behavioral performance as non-anxious individuals, although they recruit more attentional resources. In line with that, Hum et al. (2013b) also conducted a study in order to investigate the N2 before and after CBT intervention. They showed an enhancement of the N2 amplitude in children who had less anxious symptoms after therapy. This suggests that children who benefit more from CBT are those who recruit more cognitive resources while performing an attentional task. These results are also correspond the findings of other studies that showed an enhancement of prefrontal activity and of the N2 (Eldar and Bar-Haim, 2010; Maslowsky et al., 2010). However, it may seem counterintuitive that N2 enhancement can be interpreted as reflecting attentional control impairment in some studies, but improvement in others; thus, future studies are required for clarification, notably by controlling the experimental protocol and the level of conflict induced. High density recordings may allow detection of the neural source with more precision; it could be interesting to use combined fMRI and electro-encephalography (EEG) techniques (Campanella et al., 2013) to develop a precise model of the relationship between the occurrence of the N2 occurrence and the activity in the ACC and PFC.

Regarding the P3, studies have shown an enhancement of the P3 in children with anxiety disorders when they have to process and inhibit irrelevant stimuli that have a strong emotional valence (Éismont et al., 2009). These results concur agree with the hypothesis of cortical immaturity. Indeed, the major generators of the P3 are located in the PFC which is implicated in affect mediation, cognition and behavior regulation (Halgren et al., 1998). The maturation of the PFC takes longer in childhood, explaining the difficulties of anxious children in consciously controlling attentional processing and regulating their emotions. Since we here focused on the inhibitory capability of anxious children, this review mainly considered about the P3 no-go component. Nevertheless, the P3-go may also be interesting in this context. Indeed, studies conducted in anxious adults have demonstrated an enhancement of RTs for the recognition of deviant faces in attentional oddball paradigms. This enhancement was accompanied by an enhancement of the P3 amplitude suggesting facilitated attentional processing of deviant stimuli (Rossignol et al., 2005; Moser et al., 2008; Righi et al., 2009). These results recall the distinction between the notions of efficacy and the effectiveness by Eysenck et al. (2007) since the increased electro-physiological responses reveal cognitive strategies used by anxious individuals in order to maintain attentional performances.

The LPP also presented some particularities in anxious children. Indeed, Kujawa et al. (2015) showed enhancement of LPP amplitudes in these children when they were confronted with angry or fearful faces. The results suggest sustained attentionnal processing in response to threatening faces (Bar-Haim et al., 2007; Richards et al., 2014). Furthermore, as highlighted by Dennis and Chen (2007), the LPP has an excellent temporal sensitivity and thus allows detection of similarities and differences between children suffering from different disorders, and also allows comparison of child and adult performance in an emotion regulation task. The LPP also appears to be a clinical measure for determining which children are at risk of developing psychopathological conditions due to difficulties with emotional regulation. Indeed, Kujawa et al. (2012) have demonstrated that the LPP can be considered as a vulnerability marker for depression.

Finally, studies conducted in children with anxiety disorders showed an enhancement of the ERN amplitude. This enhancement was also observed in children with depressive affects (Ladouceur et al., 2006). This may be due to a disorder of the maturation of the ACC which is the generator of this component. However, the literature shows that the ACC and the PFC are the generators of attentional control (Banich et al., 2000), particularly in conflict situations (Milham et al., 2001). A maturation disorder thus has an impact on attentional control, leading to attentional biases.

The results reviewed here confirm the notion that emotional biases in children with anxiety disorders can be indexed by electro-physiological component. Furthermore, these emotional biases highlight a deficit of attentional control in these children (Eysenck et al., 2007). The attentional control deficit leads to an imbalance between the automatic attentional subsystem and the strategic attentional subsystem, so that attentional biases could be observed at different stages of the attentional processing.

**Figure 1:**
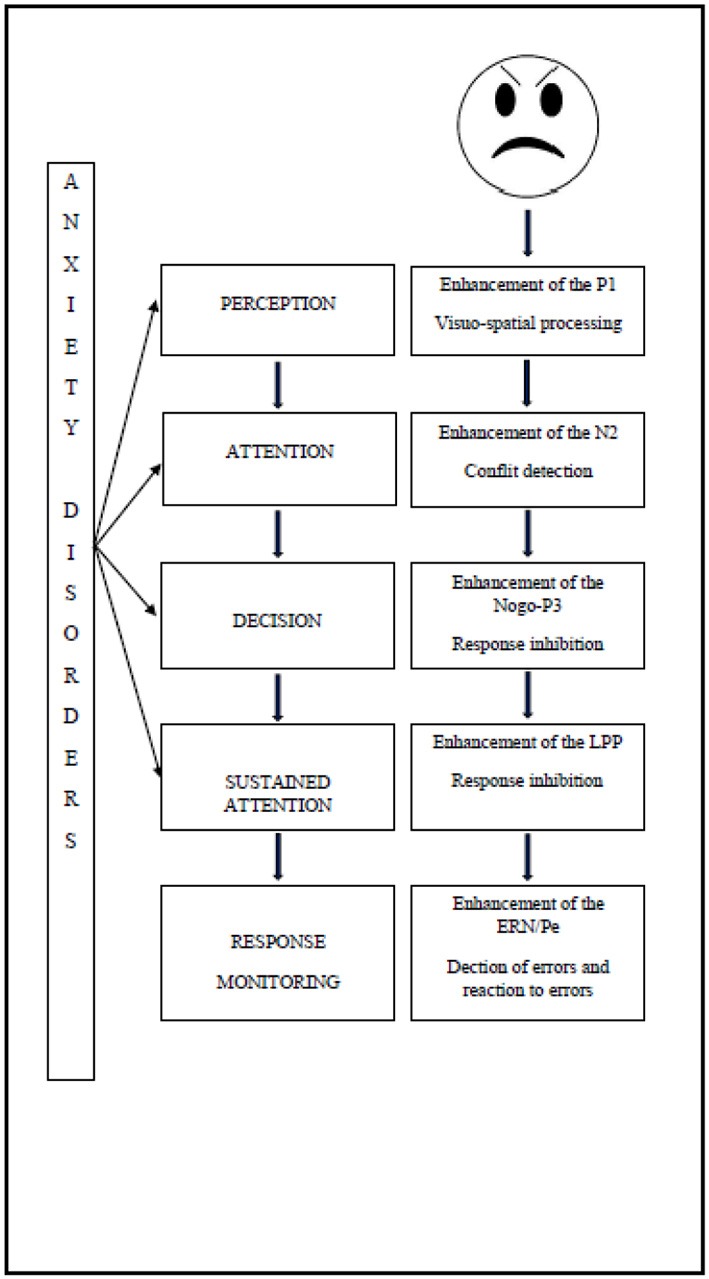
**Impact of anxiety disorder on attention processing of emotional stimuli**.

These biases can be attested by the study of the underlying electro-physiological components in children with anxiety disorders as shown in Figure 1.

The increased use of neural resources manifests immediately after the onset of the threatening stimulus. Firstly, studies have shown the impact of the attentional perception level, since the enhancement of P1 reflects visuo-spatial processing (Hum et al., 2013a). Secondly, the deficit of attentional control has an impact on conflict detection, manifested by an enhancement of the N2 (Hum et al., 2013a), and on response inhibition, manifested by enhancement of the P3 (Éismont et al., 2009). The attentional control deficit also has an impact on sustained attention, as manifested by an enhancement of the LPP amplitude in children (Kujawa et al., 2015). Finally, it has an impact during response monitoring, since studies have shown an enhancement of the ERN, thereby reflecting error detection and an enhancement of the Pe which is associated with error recognition (Ladouceur et al., 2006).

The results observed in anxious children are in line with those that had been observed in adults with the same psychopathological states (for review, see Bar-Haim et al., 2005). Furthermore, studies of the different components appear to be interesting in the early investigation of attentional biases in children who are at risk of developing a psychopathological disorder. Pollak and Tolley-Schell (2003) showed that hypervigilance to threatening faces in abused children manifested as an enhancement of the P1. The P3 was also enhanced when children were confronted with an angry expression from their mother (Shackman et al., 2007). The N2 can be modulated by extreme shyness (Henderson, 2010). Behavioral inhibition also has an impact on ERN. Indeed, Lahat et al. (2014) have stated that enhancement of the ERN in these children suggests increased vigilance and a fear of negative evaluation, which could lead to the development of anxiety disorders. These results support the hypothesis that some electro-physiological components can be considered as endophenotypes or biological markers of anxiety disorders. Early investigation of these components could lead to the development of different types of innovative care for children at risk of developing psycho-pathological disorders. Indeed, the association between an attentional control deficit and anxious symptoms due to the development of attentional biases toward threat indicates that attentional training programs may be useful in the treatment and prevention of anxiety disorders (Bar-Haim, 2010; Bar-Haim et al., 2011; Eldar et al., 2012). This paper summarized the existing literature on attentional processing of emotional information and their electro-physiological correlates. Although the literature is emergent, consistent results have been demonstrated, although further replication and specification are required. Indeed, the disctinctions between the effects of various anxiety disorders may be difficult to establish, since most of studies have not distinguish between the different types of anxiety disorders. Furthermore, subsequent studies (e.g., those on P1) are needed to determine whether the effects observed in anxious children are specific to emotional stimuli or involve all types of stimuli. Such future studies could also indicate in which type of anxiety disorder the observed effects are the most consistent. Indeed, we should compare different types of anxiety disorders and investigate whether the observed electro-physiological effects are correlated to a specific dimension of anxiety (e.g., fear of being judged) or to a more general effect of arousal.

## Conclusion

In conclusion, this review highlighted the fact that the study of electro-physiological components that underpin attentional processing of emotional stimuli in anxious children allow modeling of the impact of anxiety on cognition. Indeed, the results summarized here support the hypothesis of an attentional control deficit in anxious children, leading to attentional biases. We can observe these biases at different stages of the attentional process by studying the underpinning ERP components. However, further studies are needed in to obtain more information and provide a more comprehensive theoretical framework for this subject.

## Author contributions

MR had the initial ideas; EW conducted literature searches and wrote the first draft of the manuscript; EW and MR revised the text and both authors have approved the final manuscript.

## Author notes

The authors would like to thank Laurent Lefebvre (Umons) and Yacine Ouzzahra (University of Luxembourg) for their useful comments of a first version of this text. This work has been financed by a FRESH/FNRS grant. Authors report no actual or potential conflicts of interests.

## Funding

MR is funded by the University of Mons. EW is funded by the Fonds pour la Recherche Scientifique/Fonds National de la Recherche Scientifique.

### Conflict of interest statement

The authors declare that the research was conducted in the absence of any commercial or financial relationships that could be construed as a potential conflict of interest.

## References

[B1] Allison T. Puce A. Spencer D. D. McCarthy G. (1999). Electrophysiological studies of human face perception. I: potentials generated in occipitotemporal cortex by face and non-face stimuli. Cereb. Cortex 9, 415–430. 10.1093/cercor/9.5.415 10450888

[B2] Ansari T. L. Derakshan N. (2011a). The neural correlates of cognitive effort in anxiety: effects on processing efficiency. Biol. Psychol. 86, 337–348. 10.1016/j.biopsycho.2010.12.013 21277934

[B3] Ansari T. L. Derakshan N. (2011b). The neural correlates of impaired inhibitory control in anxiety. Neuropsychologia 49, 1146–1153. 10.1016/j.neuropsychologia.2011.01.019 21241717

[B4] Banich M. T. Milham M. P. Atchley R. A. Cohen N. J. Webb A. Wszalek T.. (2000). Prefrontal regions play a predominant role in imposing an attentional ‘set’: evidence from fMRI. Brain Res. Cogn. Brain Res. 10, 1–9. 10.1016/S0926-6410(00)00015-X 10978687

[B5] Banich M. T. Milham M. P. Jacobson B. L. Webb A. Wszalek T. Cohen N. J.. (2001). Attentional selection and the processing of task-irrelevant information: insights from fMRI examinations of the Stroop task. Prog. Brain Res. 134, 459–470. 10.1016/S0079-6123(01)34030-X 11702561

[B6] Bar-Haim Y. (2010). Research review: attention bias modification (ABM): a novel treatment for anxiety disorders. J. Child Psychol. Psychiatry 51, 859–870. 10.1111/j.1469-7610.2010.02251.x 20456540

[B7] Bar-Haim Y. Fox N. A. Benson B. Guyer A. E. Williams A. Nelson E. E.. (2009). Neural correlates of reward processing in adolescents with a history of inhibited temperament. Psychol. Sci. 20, 1009–1018. 10.1111/j.1467-9280.2009.02401.x 19594857 PMC2785902

[B8] Bar-Haim Y. Lamy D. Glickman S. (2005). Attentional bias in anxiety: a behavioral and ERP study. Brain Cogn. 59, 11–22. 10.1016/j.bandc.2005.03.005 15919145

[B9] Bar-Haim Y. Lamy D. Pergamin L. Bakermans-Kranenburg M. J. van Ijzendoorn M. H. (2007). Threat-related attentional bias in anxious and nonanxious individuals: a meta-analytic study. Psychol. Bull. 133, 1–24. 10.1037/0033-2909.133.1.1 17201568

[B10] Bar-Haim Y. Morag I. Glickman S. (2011). Training anxious children to disengage attention from threat: a randomized controlled trial. J. Child Psychol. Psychiatry 52, 861–869. 10.1111/j.1469-7610.2011.02368.x 21250993

[B11] Batty M. Taylor M. J. (2003). Early processing of the six basic facial emotional expressions. Cogn. Brain Res. 17, 613–620. 10.1016/S0926-6410(03)00174-5 14561449

[B12] Batty M. Taylor M. J. (2006). The development of emotional face processing during childhood. Dev. Sci. 9, 207–220. 10.1111/j.1467-7687.2006.00480.x 16472321

[B13] Beesdo K. Knappe S. Pine D. S. (2009). Anxiety and anxiety disorders in children and adolescents: developmental issues and implications for DSM-V. Psychiatr. Clin. North Am. 32, 483–524. 10.1016/j.psc.2009.06.002 19716988 PMC3018839

[B14] Bernstein P. S. Scheffers M. K. Coles M. G. (1995). “Where did I go wrong?” A psychophysiological analysis of error detection. J. Exp. Psychol. Hum. Percept. Perform. 21, 1312–1322. 10.1037/0096-1523.21.6.1312 7490583

[B15] Bishop S. J. (2009). Trait anxiety and impoverished prefrontal control of attention. Nat. Neurosci. 12, 92–98. 10.1038/nn.2242 19079249

[B16] Bokura H. Yamaguchi S. Kobayashi S. (2001). Electrophysiological correlates for response inhibition in a Go/NoGo task. Clin. Neurophysiol. 112, 2224–2232. 10.1016/S1388-2457(01)00691-5 11738192

[B17] Bruin K. S. Wijers A. A. van Staveren A. S. J. (2001). Response priming in a go/nogo task: do we have to explain the go/nogo N2 effect in terms of response activation instead of inhibition. Clin. Neurophysiol. 112, 1660–1671. 10.1016/S1388-2457(01)00601-0 11514249

[B18] Buss K. A. Dennis T. A. Brooker R. J. Sippel L. M. (2011). An ERP study of conflict monitoring in 4-8-year old children: associations with temperament. Dev. Cogn. Neurosci. 1, 131–140. 10.1016/j.dcn.2010.12.003 21666879 PMC3111917

[B19] Campanella S. Bourguignon M. Peigneux P. Metens T. Nouali M. Goldman S.. (2013). BOLD response to deviant face detection informed by P300 event-related potential parameters: a simultaneous ERP–fMRI study. Neuroimage 71, 92–103. 10.1016/j.neuroimage.2012.12.077 23313569

[B20] Carrasco M. Harbin S. M. Nienhuis J. K. Fitzgerald K. D. Gehring W. J. Hanna G. L. (2013). Increased error−related brain activity in youth with obsessive−compulsive disorder and unaffected siblings. Depress. Anxiety 30, 39–46. 10.1002/da.22035 23225541

[B21] Carretié L. (2014). Exogenous (automatic) attention to emotional stimuli: a review. Cogn. Affect. Behav. Neurosci. 14, 1228–1258. 10.3758/s13415-014-0270-2 24683062 PMC4218981

[B22] Corbetta M. Shulman G. L. (2002). Control of goal-directed and stimulus-driven attention in the brain. Nat. Rev. Neurosci. 3, 201–215. 10.1038/nrn755 11994752

[B23] Csibra G. Kushnerenko E. Grossmann T. (2008). Electrophysiological methods in studying infant cognitive development, in Handbook of Developmental Cognitive Neuroscience, 2nd Edn., eds Nelson C. A. Luciana M. (Cambridge, MA: MIT Press), 247–262.

[B24] Cuthbert B. N. Schupp H. T. Bradley M. M. Birbaumer N. Lang P. J. (2000). Brain potentials in affective picture processing: covariation with autonomic arousal and affective report. Biol. Psychol. 52, 95–111. 10.1016/S0301-0511(99)00044-7 10699350

[B25] Davies P. L. Segalowitz S. J. Gavin W. J. (2004). Development of error-monitoring event-related potentials in adolescents. Ann. N.Y. Acad. Sci. 1021, 324–328. 10.1196/annals.1308.039 15251904

[B26] DeCicco J. M. Solomon B. Dennis T. A. (2012). Neural correlates of cognitive reappraisal in children: an ERP study. Dev. Cogn. Neurosci. 2, 70–80. 10.1016/j.dcn.2011.05.009 22163262 PMC3234882

[B27] Dennis T. A. Chen C.-C. (2007). Emotional face processing and attention performance in three domains: neurophysiological mechanisms and moderating effects of trait anxiety. Int. J. Psychophysiol. 65, 10–19. 10.1016/j.ijpsycho.2007.02.006 17383040 PMC2001230

[B28] Dennis T. A. Hajcak G. (2009). The late positive potential: a neurophysiological marker for emotion regulation in children. J. Child Psychol. Psychiatry 50, 1373–1383. 10.1111/j.1469-7610.2009.02168.x 19754501 PMC3019134

[B29] Dunning J. P. Hajcak G. (2009). See no evil: directing visual attention within unpleasant images modulates the electrocortical response. Psychophysiology 46, 28–33. 10.1111/j.1469-8986.2008.00723.x 18992071

[B30] Éismont E. Lutsyuk N. Pavlenko V. (2009). Reflection of anxiety in the characteristics of evoked EEG potentials in 10-to 11-year-old children. Neurophysiology 41, 435–442. 10.1007/s11062-010-9123-y

[B31] Eldar S. Apter A. Lotan D. Edgar K. P. Naim R. Fox N. A.. (2012). Attention bias modification treatment for pediatric anxiety disorders: a randomized controlled trial. Am. J. Psychiatry 169, 213–220. 10.1176/appi.ajp.2011.11060886 22423353 PMC3491316

[B32] Eldar S. Bar-Haim Y. (2010). Neural plasticity in response to attention training in anxiety. Psychol. Med. 40, 667–677. 10.1017/S0033291709990766 19627649

[B33] Enriquez-Geppert S. Konrad C. Pantev C. Huster R. J. (2010). Conflict and inhibition differentially affect the N200/P300 complex in a combined go/nogo and stop-signal task. Neuroimage 51, 877–887. 10.1016/j.neuroimage.2010.02.043 20188191

[B34] Eysenck M. W. Derakshan N. Santos R. Calvo M. G. (2007). Anxiety and cognitive performance: attentional control theory. Emotion 7:336. 10.1037/1528-3542.7.2.336 17516812

[B35] Falkenstein M. Hohnsbein J. Hoormann J. Blanke L. (1991). Effects of crossmodal divided attention on late ERP components. II. Error processing in choice reaction tasks. Electroencephalogr. Clin. Neurophysiol. 78, 447–455. 10.1016/0013-4694(91)90062-9 1712280

[B36] Falkenstein M. Hoormann J. Christ S. Hohnsbein J. (2000). ERP components on reaction errors and their functional significance: a tutorial. Biol. Psychol. 51, 87–107. 10.1016/S0301-0511(99)00031-9 10686361

[B37] Falkenstein M. Hoormann J. Hohnsbein J. (1999). ERP components in Go/Nogo tasks and their relation to inhibition. Acta Psychol. 101, 267–291. 10.1016/S0001-6918(99)00008-6 10344188

[B38] Fallgatter A. J. Strik W. K. (1999). The NoGo-anteriorization as a neurophysiological standard-index for cognitive response control. Int. J. Psychophysiol. 32, 233–238. 10.1016/S0167-8760(99)00018-5 10437634

[B39] Foti D. Hajcak G. (2008). Deconstructing reappraisal: descriptions preceding arousing pictures modulate the subsequent neural response. J. Cogn. Neurosci. 20, 977–988. 10.1162/jocn.2008.20066 18211235

[B40] Foti D. Hajcak G. Dien J. (2009). Differentiating neural responses to emotional pictures: evidence from temporal-spatial PCA. Psychophysiology 46, 521–530. 10.1111/j.1469-8986.2009.00796.x 19496228

[B41] Fox E. Derakshan N. Shoker L. (2008). Trait anxiety modulates the electrophysiological indices of rapid spatial orienting towards angry faces. Neuroreport 19, 259–263. 10.1097/WNR.0b013e3282f53d2a 18303563

[B42] Frenkel T. I. Bar-Haim Y. (2011). Neural activation during the processing of ambiguous fearful facial expressions: an ERP study in anxious and nonanxious individuals. Biol. Psychol. 88, 188–195. 10.1016/j.biopsycho.2011.08.001 21846487

[B43] Goldsmith H. H. Rothbart M. K. (1999). The Laboratory Temperament Assessment Battery. Locomotor Version, 3. Eugene: Department of Psychology, University of Oregon.

[B44] Grammer J. K. Carrasco M. Gehring W. J. Morrison F. J. (2014). Age-related changes in error processing in young children: a school-based investigation. Dev. Cogn. Neurosci. 9, 93–105. 10.1016/j.dcn.2014.02.001 24631799 PMC4061373

[B45] Grasso D. J. Simons R. F. (2012). Electrophysiological responses to threat in youth with and without Posttraumatic Stress Disorder. Biol. Psychol. 90, 88–96. 10.1016/j.biopsycho.2012.02.015 22406756 PMC3319483

[B46] Hajcak G. Dennis T. A. (2009). Brain potentials during affective picture processing in children. Biol. Psychol. 80, 333–338. 10.1016/j.biopsycho.2008.11.006 19103249 PMC2656402

[B47] Hajcak G. Dunning J. P. Foti D. (2007). Neural response to emotional pictures is unaffected by concurrent task difficulty: an event-related potential study. Behav. Neurosci. 121, 1156–1162. 10.1037/0735-7044.121.6.1156 18085868

[B48] Hajcak G. MacNamara A. Foti D. Ferri J. Keil A. (2013). The dynamic allocation of attention to emotion: simultaneous and independent evidence from the late positive potential and steady state visual evoked potentials. Biol. Psychol. 92, 447–455. 10.1016/j.biopsycho.2011.11.012 22155660

[B49] Hajcak G. McDonald N. Simons R. F. (2003). Anxiety and error-related brain activity. Biol. Psychol. 64, 77–90. 10.1016/S0301-0511(03)00103-0 14602356

[B50] Hajcak G. Olvet D. M. (2008). The persistence of attention to emotion: brain potentials during and after picture presentation. Emotion 8, 250–255. 10.1037/1528-3542.8.2.250 18410198

[B51] Halgren E. Marinkovic K. Chauvel P. (1998). Generators of the late cognitive potentials in auditory and visual oddball tasks. Electroencephalogr. Clin. Neurophysiol. 106, 156–164. 10.1016/S0013-4694(97)00119-3 9741777

[B52] Henderson H. A. (2010). Electrophysiological correlates of cognitive control and the regulation of shyness in children. Dev. Neuropsychol. 35, 177–193. 10.1080/87565640903526538 20390601

[B53] Hoehl S. Striano T. (2010). The development of emotional face and eye gaze processing. Dev. Sci. 13, 813–825. 10.1111/j.1467-7687.2009.00944.x 20977553

[B54] Hogan A. M. Vargha-Khadem F. Kirkham F. J. Baldeweg T. (2005). Maturation of action monitoring from adolescence to adulthood: an ERP study. Dev. Sci. 8, 525–534. 10.1111/j.1467-7687.2005.00444.x 16246244

[B55] Holmes A. Nielsen M. K. Tipper S. Green S. (2009). An electrophysiological investigation into the automaticity of emotional face processing in high versus low trait anxious individuals. Cogn. Affect. Behav. Neurosci. 9, 323–334. 10.3758/CABN.9.3.323 19679767

[B56] Holroyd K. J. Martinati L. C. Trabetti E. Scherpbier T. Eleff S. M. Boner A. L.. (1998). Asthma and bronchial hyperresponsiveness linked to the XY long arm pseudoautosomal region. Genomics 52, 233–235. 10.1006/geno.1998.5445 9782093

[B57] Hua M. Han Z. R. Zhou R. (2015). Cognitive reappraisal in preschoolers: neuropsychological evidence of emotion regulation from an ERP study. Dev. Neuropsychol. 40, 279–290. 10.1080/87565641.2015.1069827 26230638

[B58] Hum K. M. Manassis K. Lewis M. D. (2013a). Neural mechanisms of emotion regulation in childhood anxiety. J. Child Psychol. Psychiatry 54, 552–564. 10.1111/j.1469-7610.2012.02609.x 23046115

[B59] Hum K. M. Manassis K. Lewis M. D. (2013b). Neurophysiological markers that predict and track treatment outcomes in childhood anxiety. J. Abnorm. Child Psychol. 41, 1243–1255. 10.1007/s10802-013-9755-7 23690280

[B60] James A. C. James G. Cowdrey F. A. Soler A. Choke A. (2013). Cognitive behavioural therapy for anxiety disorders in children and adolescents. Cochrane Database Syst. Rev. 6:CD004690. 10.1002/14651858.CD004690.pub3 23733328

[B61] Jessen S. Grossmann T. (2016). The developmental emergence of unconscious fear processing from eyes during infancy. J. Exp. Child Psychol. 142, 334–343. 10.1016/j.jecp.2015.09.009 26493612

[B62] Jonkman L. Lansbergen M. Stauder J. (2003). Developmental differences in behavioral and event-related brain responses associated with response preparation and inhibition in a go/nogo task. Psychophysiology 40, 752–761. 10.1111/1469-8986.00075 14696728

[B63] Jonkman L. Sniedt F. Kemner C. (2007). Source localization of the Nogo-N2: a developmental study. Clin. Neurophysiol. 118, 1069–1077. 10.1016/j.clinph.2007.01.017 17368096

[B64] Jonkman L. M. (2006). The development of preparation, conflict monitoring and inhibition from early childhood to young adulthood; a Go/Nogo ERP study. Brain Res. 1097, 181–193. 10.1016/j.brainres.2006.04.064 16729977

[B65] Kiehl K. A. Liddle P. F. Hopfinger J. B. (2000). Error processing and the rostral anterior cingulate: an event-related fMRI study. Psychophysiology 37, 216–223. 10.1111/1469-8986.3720216 10731771

[B66] Kok A. (1997). Event-related-potential (ERP) reflections of mental resources: a review and synthesis. Biol. Psychol. 45, 19–56. 10.1016/S0301-0511(96)05221-0 9083643

[B67] Kujawa A. Klein D. N. Hajcak G. (2012). Electrocortical reactivity to emotional images and faces in middle childhood to early adolescence. Dev. Cogn. Neurosci. 2, 458–467. 10.1016/j.dcn.2012.03.005 22521707 PMC3404214

[B68] Kujawa A. Klein D. N. Proudfit G. H. (2013). Two-year stability of the late positive potential across middle childhood and adolescence. Biol. Psychol. 94, 290–296. 10.1016/j.biopsycho.2013.07.002 23872165 PMC3797196

[B69] Kujawa A. MacNamara A. Fitzgerald K. D. Monk C. S. Phan K. L. (2015). Enhanced neural reactivity to threatening faces in anxious youth: evidence from event-related potentials. J. Abnorm. Child Psychol. 43, 1493–1501. 10.1007/s10802-015-0029-4 25943264 PMC4751035

[B70] Ladouceur C. D. Dahl R. E. Birmaher B. Axelson D. A. Ryan N. D. (2006). Increased error−related negativity (ERN) in childhood anxiety disorders: ERP and source localization. J. Child Psychol. Psychiatry 47, 1073–1082. 10.1111/j.1469-7610.2006.01654.x 17073986

[B71] Ladouceur C. D. Dahl R. E. Carter C. S. (2004). ERP correlates of action monitoring in adolescence. Ann. N.Y. Acad. Sci. 1021, 329–336. 10.1196/annals.1308.040 15251905

[B72] Lahat A. Lamm C. Chronis-Tuscano A. Pine D. S. Henderson H. A. Fox N. A. (2014). Early behavioral inhibition and increased error monitoring predict later social phobia symptoms in childhood. J. Am. Acad. Child Adolesc. Psychiatry 53, 447–455. 10.1016/j.jaac.2013.12.019 24655654 PMC4323582

[B73] Lamm C. Walker O. L. Degnan K. A. Henderson H. A. Pine D. S. McDermott J. M.. (2014). Cognitive control moderates early childhood temperament in predicting social behavior in 7-year-old children: an ERP study. Dev. Sci. 17, 667–681. 10.1111/desc.12158 24754610 PMC4334573

[B74] Lamm C. Zelazo P. D. Lewis M. D. (2006). Neural correlates of cognitive control in childhood and adolescence: disentangling the contributions of age and executive function. Neuropsychologia 44, 2139–2148. 10.1016/j.neuropsychologia.2005.10.013 16310813

[B75] Lang P. Bradley M. M. (2007). The International Affective Picture System (IAPS) in the study of emotion and attention, in Handbook of Emotion Elicitation and Assessment, eds Davidson R. J. Ekman P. Scherer K. (New York, NY), 29–46.

[B76] Leutgeb V. Schäfer A. Köchel A. Scharmüller W. Schienle A. (2010). Psychophysiology of spider phobia in 8-to 12-year-old girls. Biol. Psychol. 85, 424–431. 10.1016/j.biopsycho.2010.09.004 20851734

[B77] Lewis M. D. Lamm C. Segalowitz S. J. Stieben J. Zelazo P. D. (2006). Neurophysiological correlates of emotion regulation in children and adolescents. J. Cogn. Neurosci. 18, 430–443. 10.1162/jocn.2006.18.3.430 16513007

[B78] Lewis M. D. Stieben J. (2004). Emotion regulation in the brain: conceptual issues and directions for developmental research. Child Dev. 75, 371–376. 10.1111/j.1467-8624.2004.00680.x 15056193

[B79] Liu Y. Huang H. McGinnis-Deweese M. Keil A. Ding M. (2012). Neural substrate of the late positive potential in emotional processing. J. Neurosci. 32, 14563–14572. 10.1523/JNEUROSCI.3109-12.2012 23077042 PMC3516184

[B80] Lonigan C. J. Phillips B. M. (2001). Temperamental influences on the development of anxiety disorders, in The Developmental Psychopathology of Anxiety, eds Vasey M. W. Dadds M. R. (New York, NY: Oxford University Press), 60–91.

[B81] Lonigan C. J. Vasey M. W. (2009). Negative affectivity, effortful control, and attention to threat-relevant stimuli. J. Abnorm. Child Psychol. 37, 387–399. 10.1007/s10802-008-9284-y 19043783

[B82] Lonigan C. J. Vasey M. W. Phillips B. M. Hazen R. A. (2004). Temperament, anxiety, and the processing of threat-relevant stimuli. J. Clin. Child Adolesc. Psychol. 33, 8–20. 10.1207/S15374424JCCP3301_2 15028537

[B83] Luck S. J. Woodman G. F. Vogel E. K. (2000). Event-related potential studies of attention. Trends Cogn. Sci. 4, 432–440. 10.1016/S1364-6613(00)01545-X 11058821

[B84] Luijten M. Machielsen M. W. J. Veltman D. J. Hester R. de Haan L. Franken I. H. A. (2014). Systematic review of ERP and fMRI studies investigating inhibitory control and error processing in people with substance dependence and behavioural addictions. J. Psychiatry Neurosci. 39, 149–169. 10.1503/jpn.130052 24359877 PMC3997601

[B85] Luu P. Tucker D. M. Derryberry D. Reed M. Poulsen C. (2003). Electrophysiological responses to errors and feedback in the process of action regulation. Psychol. Sci. 14, 47–53. 10.1111/1467-9280.01417 12564753

[B86] Luu P. Tucker D. M. Makeig S. (2004). Frontal midline theta and the error-related negativity: neurophysiological mechanisms of action regulation. Clin. Neurophysiol. 115, 1821–1835. 10.1016/j.clinph.2004.03.031 15261861

[B87] MacNamara A. Foti D. Hajcak G. (2009). Tell me about it: neural activity elicited by emotional pictures and preceding descriptions. Emotion 9, 531–543. 10.1037/a0016251 19653776

[B88] MacNamara A. Post D. Kennedy A. E. Rabinak C. A. Phan K. L. (2013). Electrocortical processing of social signals of threat in combat-related post-traumatic stress disorder. Biol. Psychol. 94, 441–449. 10.1016/j.biopsycho.2013.08.009 24025760

[B89] MacNamara A. Verges A. Kujawa A. Fitzgerald K. D. Monk C. S. Phan K. L. (2016). Age-related changes in emotional face processing across childhood and into young adulthood: evidence from event-related potentials. Dev. Psychobiol. 58, 27–38. 10.1002/dev.21341 26220144 PMC4857589

[B90] Maslowsky J. Mogg K. Bradley B. P. McClure-Tone E. Ernst M. Pine D. S.. (2010). A preliminary investigation of neural correlates of treatment in adolescents with generalized anxiety disorder. J. Child Adolesc. Psychopharmacol. 20, 105–111. 10.1089/cap.2009.0049 20415605 PMC2865364

[B91] Meyer A. Hajcak G. Torpey D. C. Kujawa A. Kim J. Bufferd S.. (2013). Increased error-related brain activity in six-year-old children with clinical anxiety. J. Abnorm. Child Psychol. 41, 1257–1266. 10.1007/s10802-013-9762-8 23700171 PMC5274547

[B92] Meyer A. Hajcak G. Torpey-Newman D. C. Kujawa A. Klein D. N. (2015). Enhanced error-related brain activity in children predicts the onset of anxiety disorders between the ages of 6 and 9. J. Abnorm. Psychol. 124, 266. 10.1037/abn0000044 25643204 PMC4428914

[B93] Meyer A. Proudfit G. H. Bufferd S. J. Kujawa A. J. Laptook R. S. Torpey D. C.. (2014). Self-reported and observed punitive parenting prospectively predicts increased error-related brain activity in six-year-old children. J. Abnorm. Child Psychol. 43, 821–829. 10.1007/s10802-014-9918-1 25092483 PMC5302091

[B94] Meyer A. Weinberg A. Klein D. N. Hajcak G. (2012). The development of the error-related negativity (ERN) and its relationship with anxiety: evidence from 8 to 13 year-olds. Dev. Cogn. Neurosci. 2, 152–161. 10.1016/j.dcn.2011.09.005 22308177 PMC3269914

[B95] Michalowski J. M. Melzig C. A. Weike A. I. Stockburger J. Schupp H. T. Hamm A. O. (2009). Brain dynamics in spider-phobic individuals exposed to phobia-relevant and other emotional stimuli. Emotion 9, 306–315. 10.1037/a0015550 19485608

[B96] Milham M. P. Banich M. T. Webb A. Barad V. Cohen N. J. Wszalek T.. (2001). The relative involvement of anterior cingulate and prefrontal cortex in attentional control depends on nature of conflict. Brain Res. Cogn. Brain Res. 12, 467–473. 10.1016/S0926-6410(01)00076-3 11689307

[B97] Moser J. S. Huppert J. D. Duval E. Simons R. F. (2008). Face processing biases in social anxiety: an electrophysiological study. Biol. Psychol. 78, 93–103. 10.1016/j.biopsycho.2008.01.005 18353522

[B98] Mueller E. M. Hofmann S. G. Santesso D. L. Meuret A. E. Bitran S. Pizzagalli D. A. (2009). Electrophysiological evidence of attentional biases in social anxiety disorder. Psychol. Med. 39, 1141–1152. 10.1017/S0033291708004820 19079826 PMC3204217

[B99] Muhlberger A. Wieser M. J. Herrmann M. J. Weyers P. Troger C. Pauli P. (2009). Early cortical processing of natural and artificial emotional faces differs between lower and higher socially anxious persons. J. Neural Transm. 116, 735–746. 10.1007/s00702-008-0108-6 18784899

[B100] Muris P. van Brakel A. M. L. Arntz A. Schouten E. (2011). Behavioral inhibition as a risk factor for the development of childhood anxiety disorders: a longitudinal study. J. Child Fam. Stud. 20, 157–170. 10.1007/s10826-010-9365-8 21475710 PMC3048305

[B101] Nelson C. A. Nugent K. M. (1990). Recognition memory and resource allocation as revealed by children's event-related potentials response to happy and angry faces. Dev. Psychol. 26:171 10.1037/0012-1649.26.2.171

[B102] Nieuwenhuis S. Ridderinkhof K. R. Blom J. Band G. P. Kok A. (2001). Error-related brain potentials are differentially related to awareness of response errors: evidence from an antisaccade task. Psychophysiology 38, 752–760. 10.1111/1469-8986.3850752 11577898

[B103] Nieuwenhuis S. Yeung N. Van Den Wildenberg W. Ridderinkhof K. R. (2003). Electrophysiological correlates of anterior cingulate function in a go/no-go task: effects of response conflict and trial type frequency. Cogn. Affect. Behav. Neurosci. 3, 17–26. 10.3758/CABN.3.1.17 12822595

[B104] Okazaki S. Hosokawa M. Kawakubo Y. Ozaki H. Maekawa H. Futakami S. (2004). Developmental change of neurocognitive motor behavior in a continuous performance test with different interstimulus intervals. Clin. Neurophysiol. 115, 1104–1113. 10.1016/j.clinph.2003.12.021 15066536

[B105] Olofsson J. K. Nordin S. Sequeira H. Polich J. (2008). Affective picture processing: an integrative review of ERP findings. Biol. Psychol. 77, 247–265. 10.1016/j.biopsycho.2007.11.006 18164800 PMC2443061

[B106] Osinsky R. Gebhardt H. Alexander N. Hennig J. (2012). Trait anxiety and the dynamics of attentional control. Biol. Psychol. 89, 252–259. 10.1016/j.biopsycho.2011.10.016 22044800

[B107] Pailing P. E. Segalowitz S. J. (2004). The effects of uncertainty in error monitoring on associated ERPs. Brain Cogn. 56, 215–233. 10.1016/j.bandc.2004.06.005 15518937

[B108] Peltola M. J. Leppänen J. M. Mäki S. Hietanen J. K. (2009). Emergence of enhanced attention to fearful faces between 5 and 7 months of age. Soc. Cogn. Affect. Neurosci. 4, 134–142. 10.1093/scan/nsn046 19174536 PMC2686224

[B109] Peschard V. Philippot P. Joassin F. Rossignol M. (2013). The impact of the stimulus features and task instructions on facial processing in social anxiety: an ERP investigation. Biol. Psychol. 93, 88–96. 10.1016/j.biopsycho.2013.01.009 23384510

[B110] Pollak S. D. Cicchetti D. Hornung K. Reed A. (2000). Recognizing emotion in faces: developmental effects of child abuse and neglect. Dev. Psychol. 36, 679–688. 10.1037/0012-1649.36.5.679 10976606

[B111] Pollak S. D. Tolley-Schell S. A. (2003). Selective attention to facial emotion in physically abused children. J. Abnorm. Psychol. 112, 323–338. 10.1037/0021-843X.112.3.323 12943012

[B112] Posner M. I. Petersen S. E. (1990). The attention system of the human brain. Annu. Rev. Neurosci. 13, 25–42. 10.1146/annurev.ne.13.030190.000325 2183676

[B113] Posner M. I. Rothbart M. K. (2007). Research on attention networks as a model for the integration of psychological science. Annu. Rev. Psychol. 58, 1–23. 10.1146/annurev.psych.58.110405.085516 17029565

[B114] Puliafico A. C. Kendall P. C. (2006). Threat-related attentional bias in anxious youth: a review. Clin. Child Fam. Psychol. Rev. 9, 162–180. 10.1007/s10567-006-0009-x 17053961

[B115] Richards H. J. Benson V. Donnelly N. Hadwin J. A. (2014). Exploring the function of selective attention and hypervigilance for threat in anxiety. Clin. Psychol. Rev. 34, 1–13. 10.1016/j.cpr.2013.10.006 24286750

[B116] Righi S. Mecacci L. Viggiano M. P. (2009). Anxiety, cognitive self-evaluation and performance: ERP correlates. J. Anxiety Disord. 23, 1132–1138. 10.1016/j.janxdis.2009.07.018 19695828

[B117] Rossignol M. Philippot P. Bissot C. Rigoulot S. Campanella S. (2012). Electrophysiological correlates of enhanced perceptual processes and attentional capture by emotional faces in social anxiety. Brain Res. 1460, 50–62. 10.1016/j.brainres.2012.04.034 22592075

[B118] Rossignol M. Philippot P. Douilliez C. Crommelinck M. Campanella S. (2005). The perception of fearful and happy facial expression is modulated by anxiety: an event-related potential study. Neurosci. Lett. 377, 115–120. 10.1016/j.neulet.2004.11.091 15740848

[B119] Santesso D. L. Segalowitz S. J. Schmidt L. A. (2005). ERP correlates of error monitoring in 10-year olds are related to socialization. Biol. Psychol. 70, 79–87. 10.1016/j.biopsycho.2004.12.004 16168252

[B120] Santesso D. L. Segalowitz S. J. Schmidt L. A. (2006). Error-related electrocortical responses are enhanced in children with obsessive–compulsive behaviors. Dev. Neuropsychol. 29, 431–445. 10.1207/s15326942dn2903_3 16671860

[B121] Sass S. M. Heller W. Stewart J. L. Silton R. L. Edgar J. C. Fisher J. E.. (2010). Time course of attentional bias in anxiety: emotion and gender specificity. Psychophysiology 47, 247–259. 10.1111/j.1469-8986.2009.00926.x 19863758 PMC3073148

[B122] Scheffers M. K. Coles M. G. Bernstein P. Gehring W. J. Donchin E. (1996). Event−related brain potentials and error−related processing: an analysis of incorrect responses to go and no−go stimuli. Psychophysiology 33, 42–53. 10.1111/j.1469-8986.1996.tb02107.x 8570794

[B123] Segalowitz S. J. Santesso D. L. Jetha M. K. (2010). Electrophysiological changes during adolescence: a review. Brain Cogn. 72, 86–100. 10.1016/j.bandc.2009.10.003 19914761

[B124] Shackman J. E. Shackman A. J. Pollak S. D. (2007). Physical abuse amplifies attention to threat and increases anxiety in children. Emotion 7:838. 10.1037/1528-3542.7.4.838 18039053

[B125] Solomon B. DeCicco J. M. Dennis T. A. (2012). Emotional picture processing in children: an ERP study. Dev. Cogn. Neurosci. 2, 110–119. 10.1016/j.dcn.2011.04.002 22163263 PMC3234883

[B126] Taylor M. J. Batty M. Itier R. J. (2004). The faces of development: a review of early face processing over childhood. J. Cogn. Neurosci. 16, 1426–1442. 10.1162/0898929042304732 15509388

[B127] Telzer E. H. Mogg K. Bradley B. P. Mai X. Ernst M. Pine D. S.. (2008). Relationship between trait anxiety, prefrontal cortex, and attention bias to angry faces in children and adolescents. Biol. Psychol. 79, 216–222. 10.1016/j.biopsycho.2008.05.004 18599179 PMC2574721

[B128] Todd R. M. Lewis M. D. Meusel L.-A. Zelazo P. D. (2008). The time course of social-emotional processing in early childhood: ERP responses to facial affect and personal familiarity in a Go-Nogo task. Neuropsychologia 46, 595–613. 10.1016/j.neuropsychologia.2007.10.011 18061633

[B129] Van Ameringen M. Mancini C. Oakman J. M. (1998). The relationship of behavioral inhibition and shyness to anxiety disorder. J. Nerv. Ment. Dis. 186, 425–431. 10.1097/00005053-199807000-00007 9680044

[B130] Van Veen V. Carter C. S. (2002). The timing of action-monitoring processes in the anterior cingulate cortex. J. Cogn. Neurosci. 14, 593–602. 10.1162/08989290260045837 12126500

[B131] Weinberg A. Riesel A. Hajcak G. (2012). Integrating multiple perspectives on error-related brain activity: the ERN as a neural indicator of trait defensive reactivity. Motiv. Emot. 36, 84–100. 10.1007/s11031-011-9269-y

[B132] Weisbrod M. Kiefer M. Marzinzik F. Spitzer M. (2000). Executive control is disturbed in schizophrenia: evidence from event-related potentials in a Go/NoGo task. Biol. Psychiatry 47, 51–60. 10.1016/S0006-3223(99)00218-8 10650449

[B133] Wessing I. Rehbein M. A. Romer G. Achtergarde S. Dobel C. Zwitserlood P.. (2015). Cognitive emotion regulation in children: reappraisal of emotional faces modulates neural source activity in a frontoparietal network. Dev. Cogn. Neurosci. 13, 1–10. 10.1016/j.dcn.2015.01.012 25796042 PMC6989777

[B134] Wiersema J. R. van der Meere J. J. Roeyers H. (2005). ERP correlates of impaired error monitoring in children with ADHD. J. Neural Transm. 112, 1417–1430. 10.1007/s00702-005-0276-6 15726277

[B135] Wiersema J. R. van der Meere J. J. Roeyers H. (2007). Developmental changes in error monitoring: an event-related potential study. Neuropsychologia 45, 1649–1657. 10.1016/j.neuropsychologia.2007.01.004 17303199

